# Supplementary material for: Calcium-dependent pathway as a primary cause of hypoxic RGC damage in monkey retinal explants
Source: PLoS One. 2025 Jul 11;20(7):e0327246. doi: 10.1371/journal.pone.0327246 (PMC12250347; doi:10.1371/journal.pone.0327246)

Original blot images of Fig1E

α-spectrin

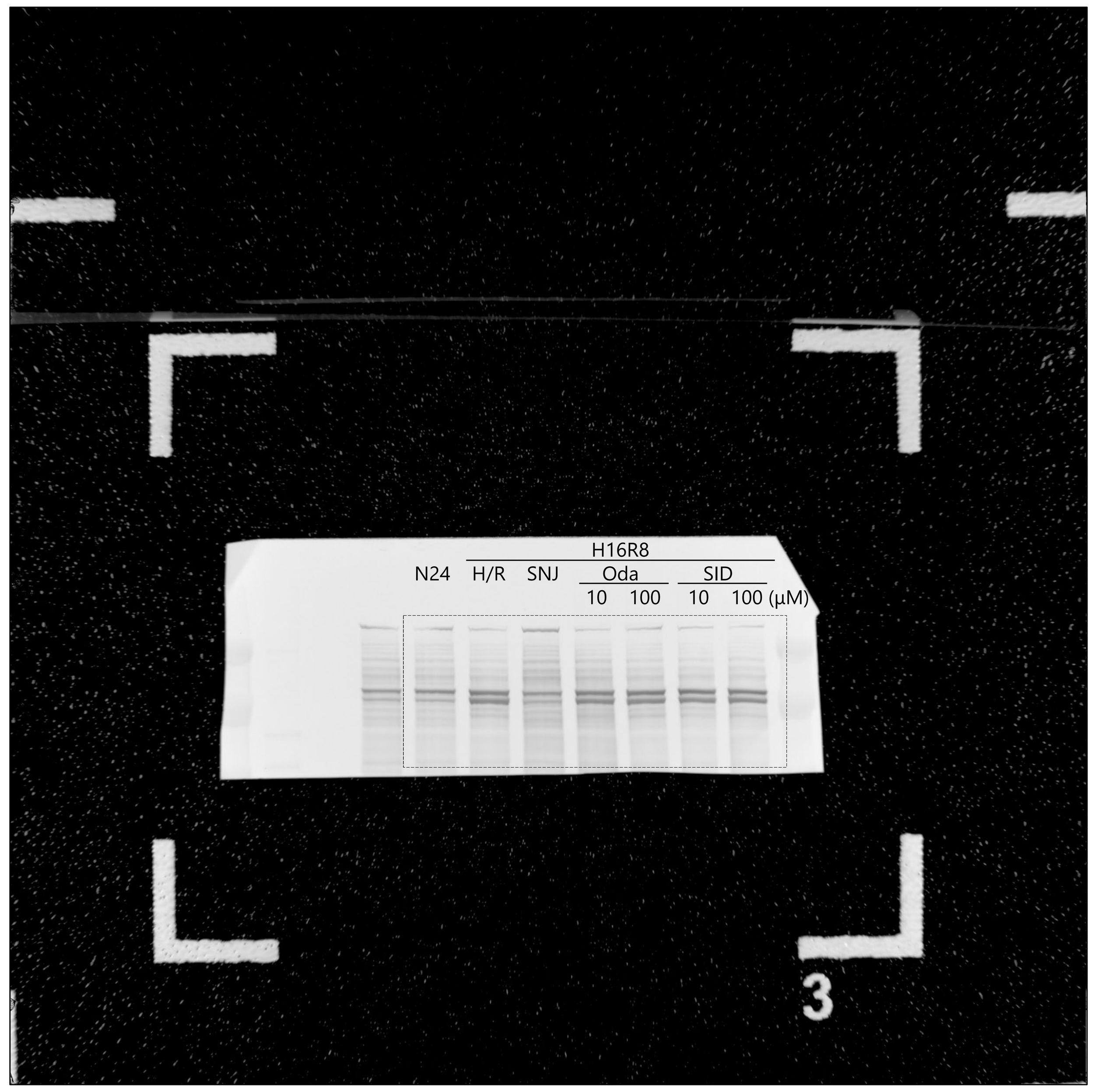

SBDP150

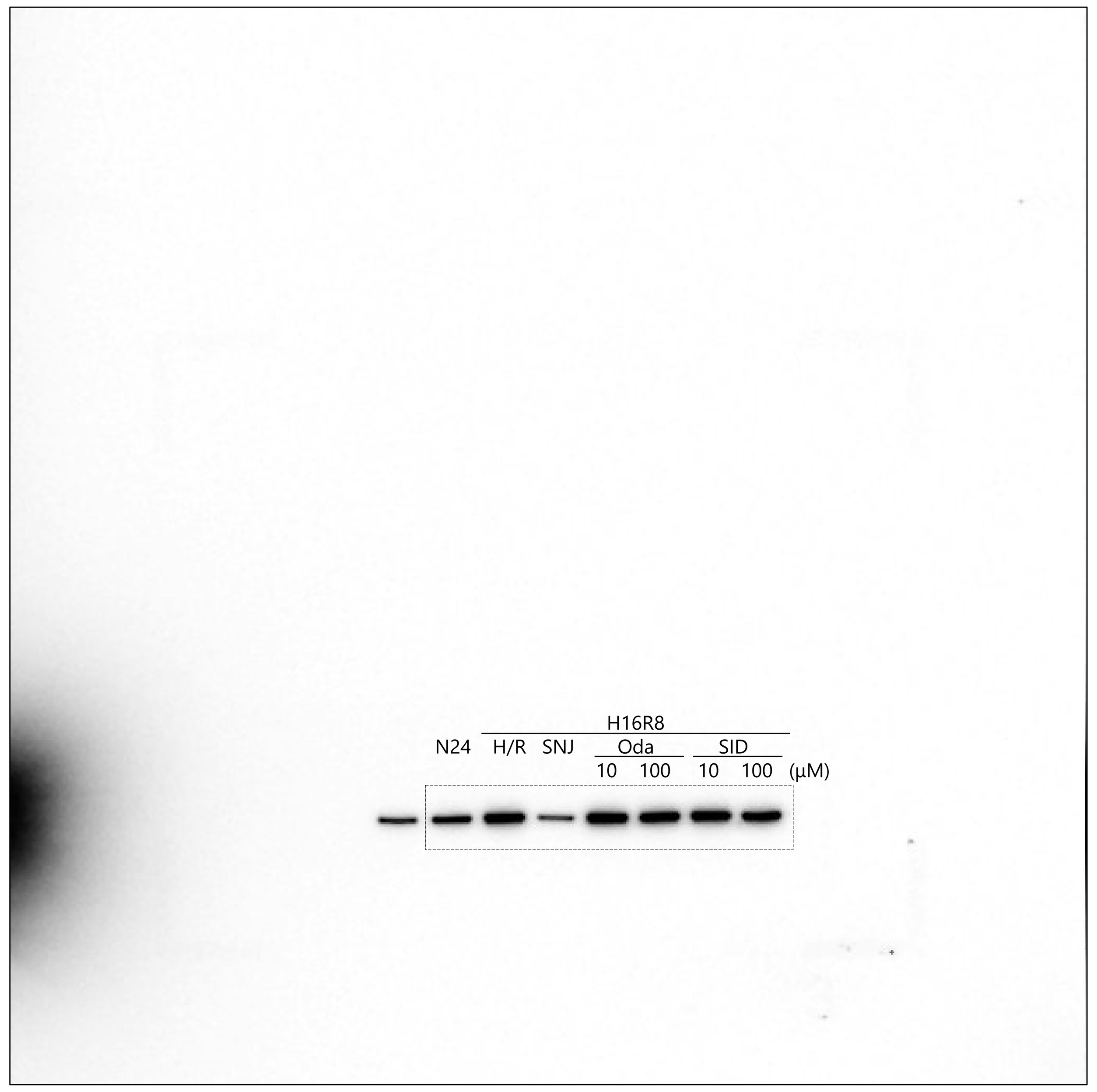

Calpain1

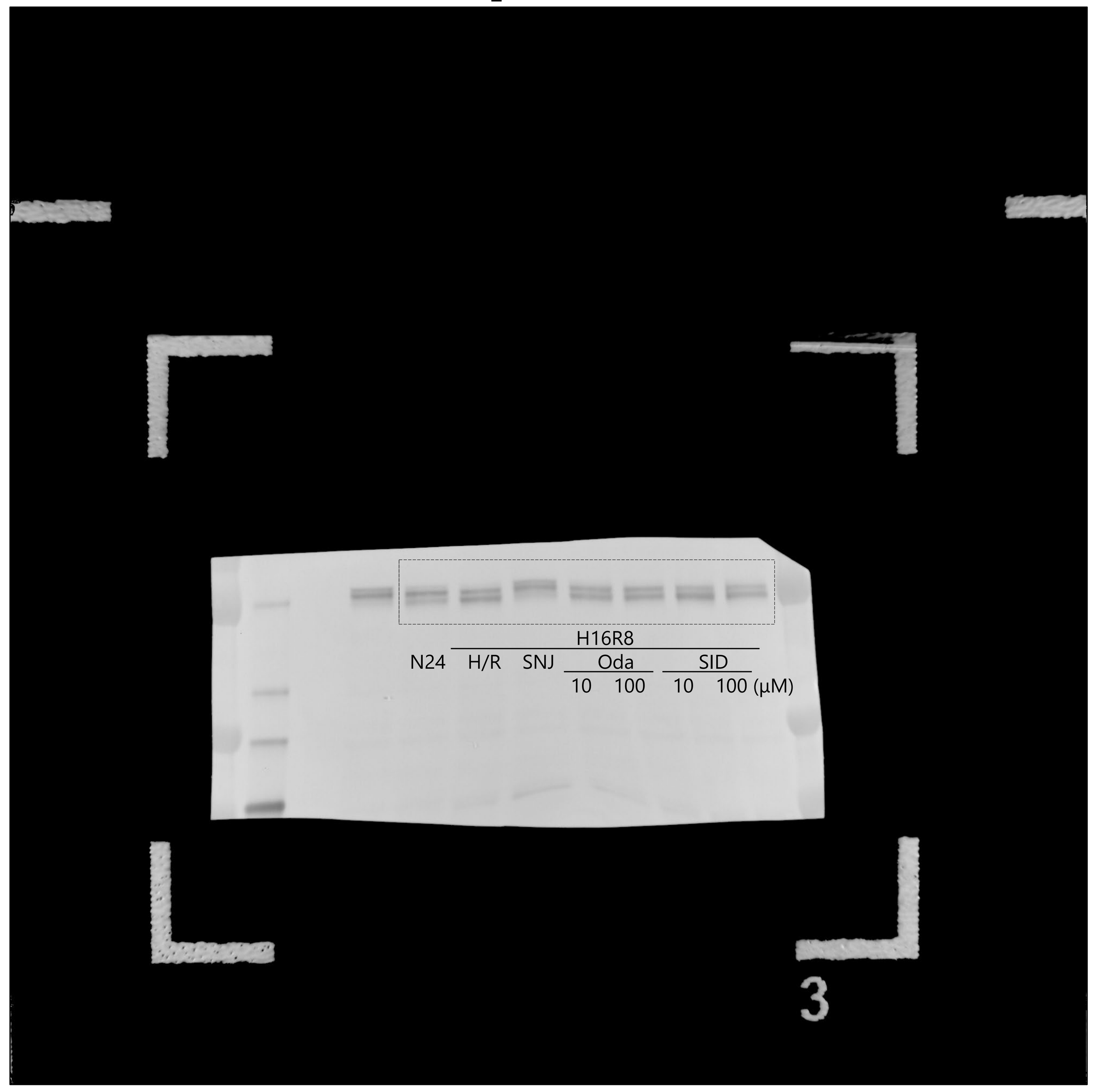

β-actin

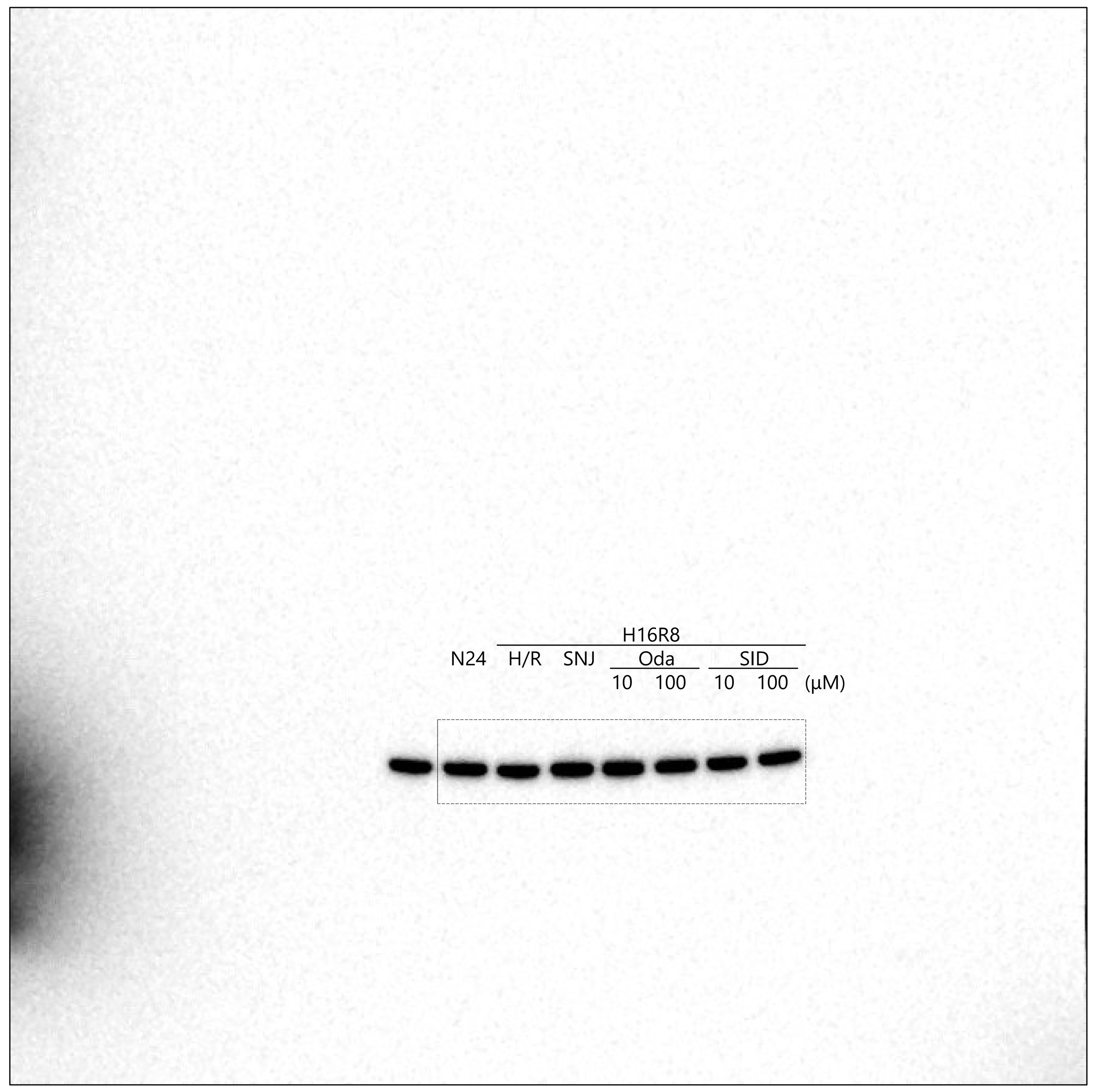

Original blot images of Fig1F

Cathepsin K

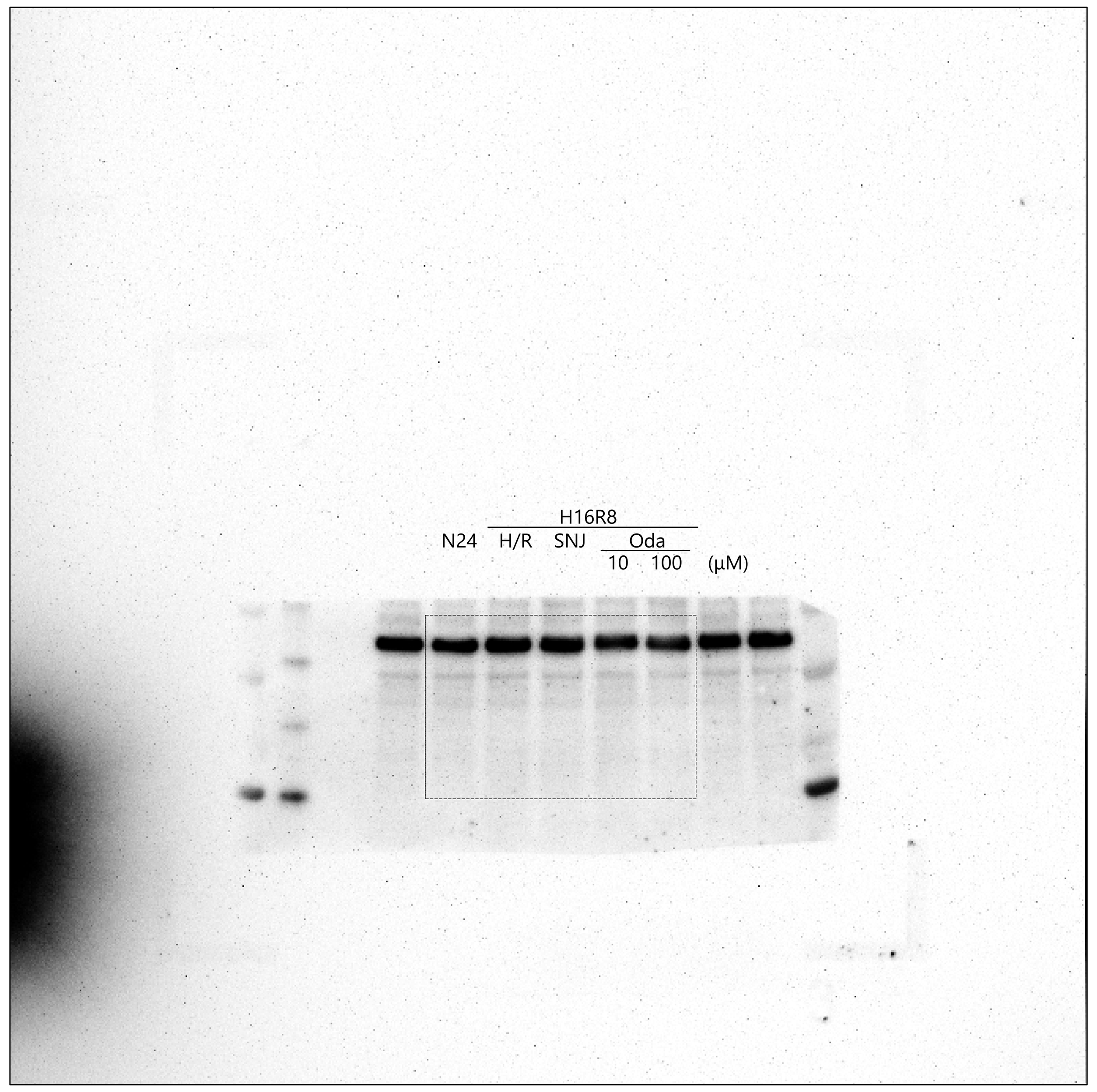

β-actin

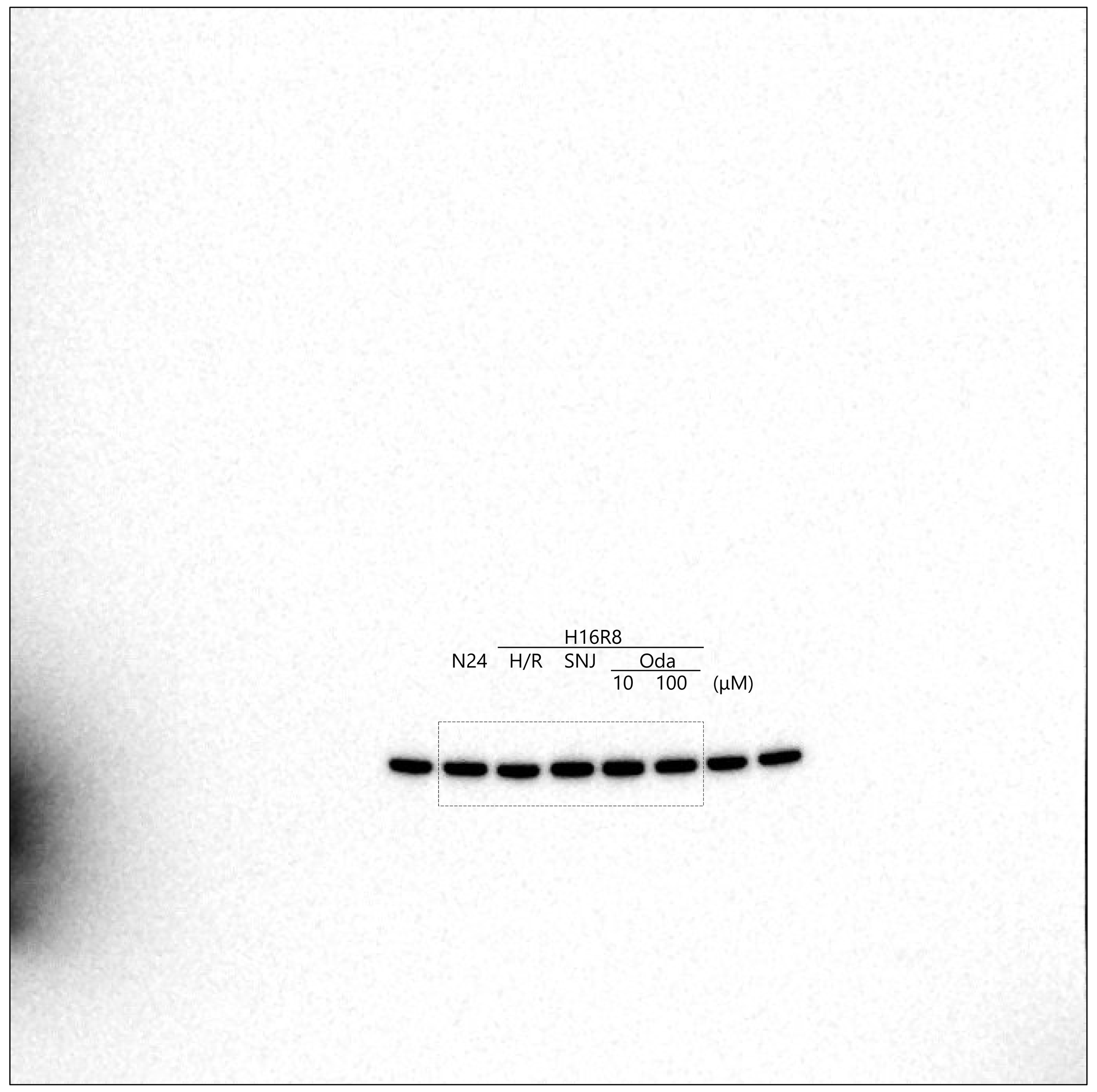

Original blot images of Fig2D

α-spectrin

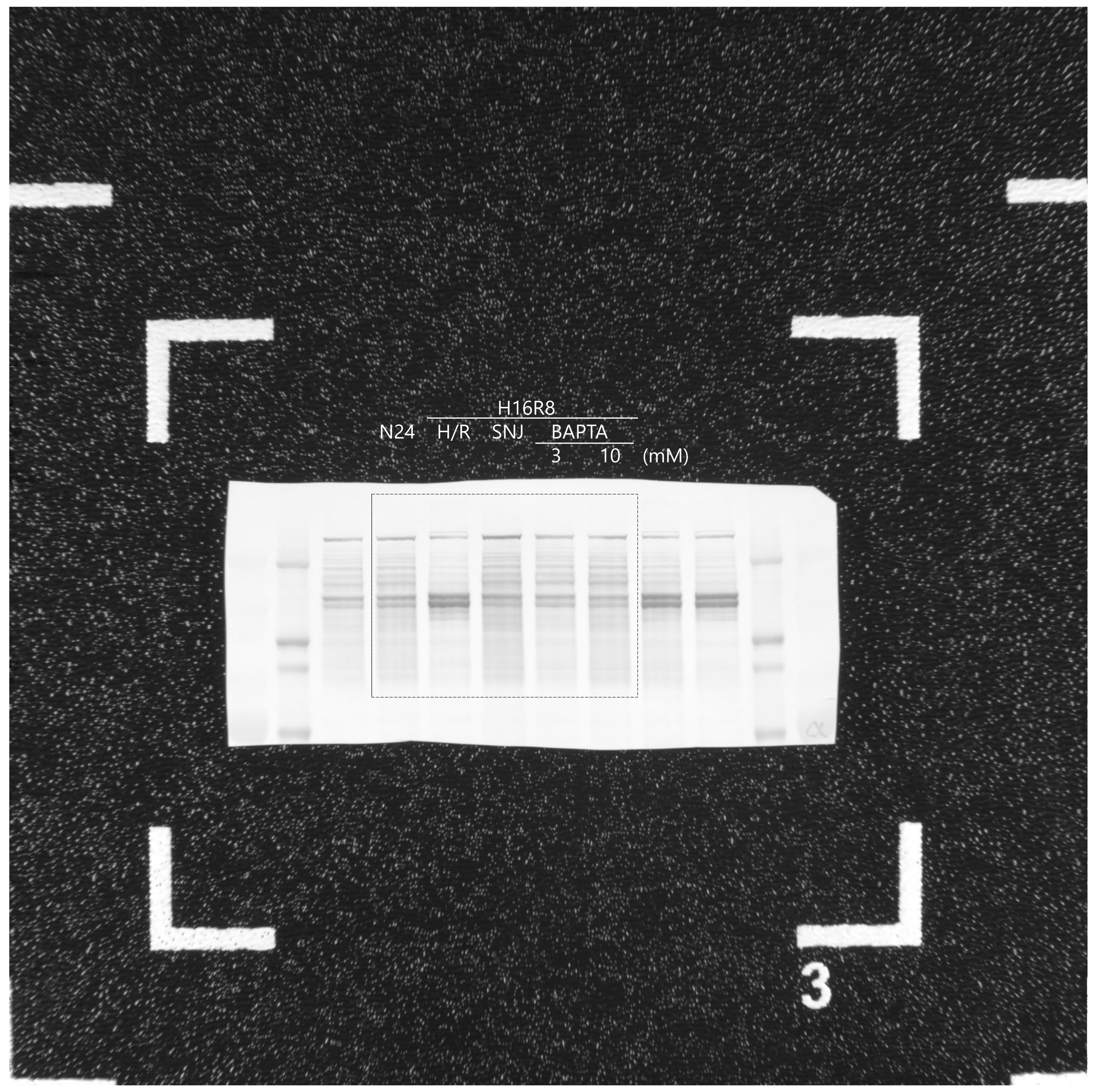

SBDP150

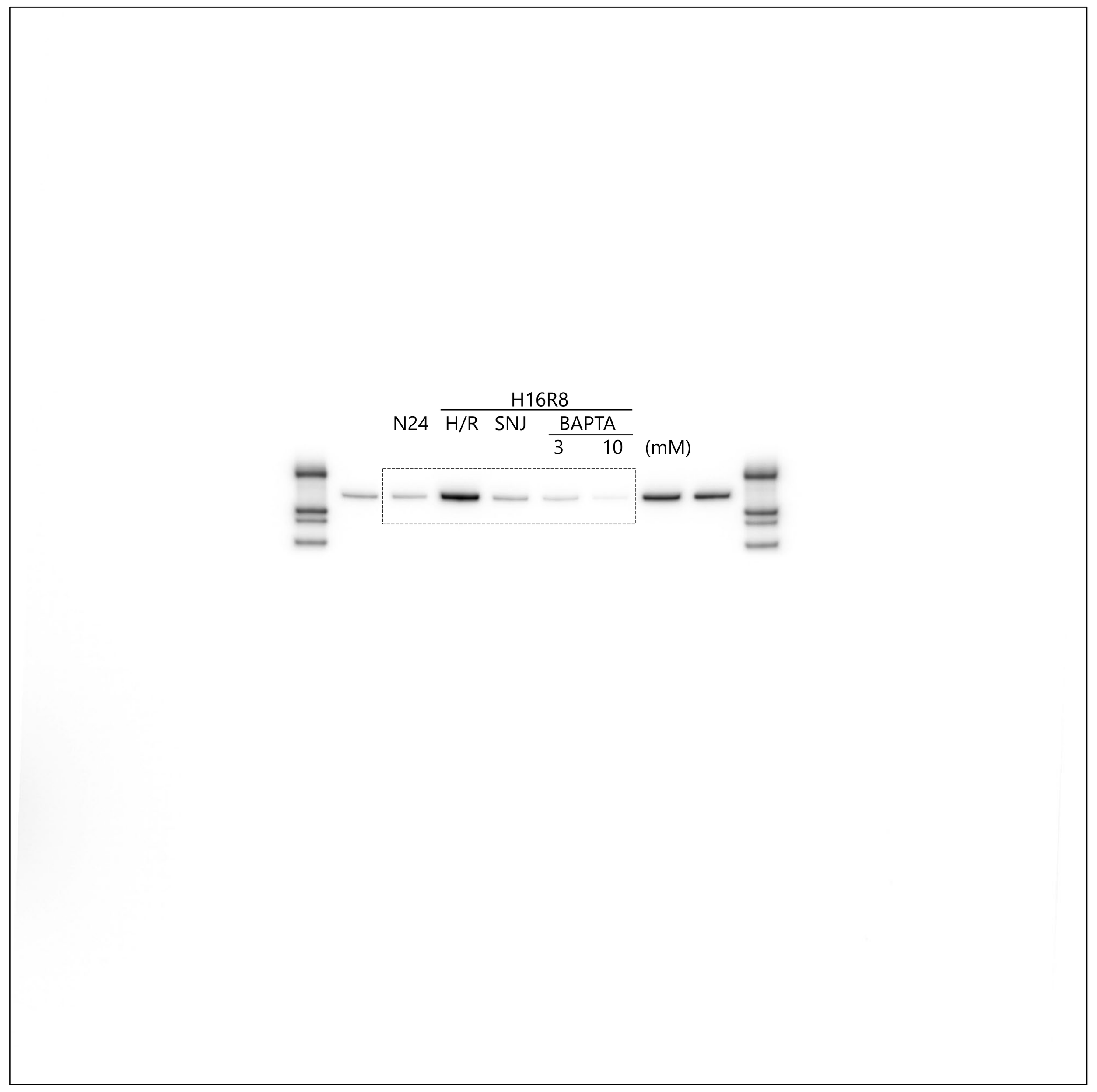

Calpain1

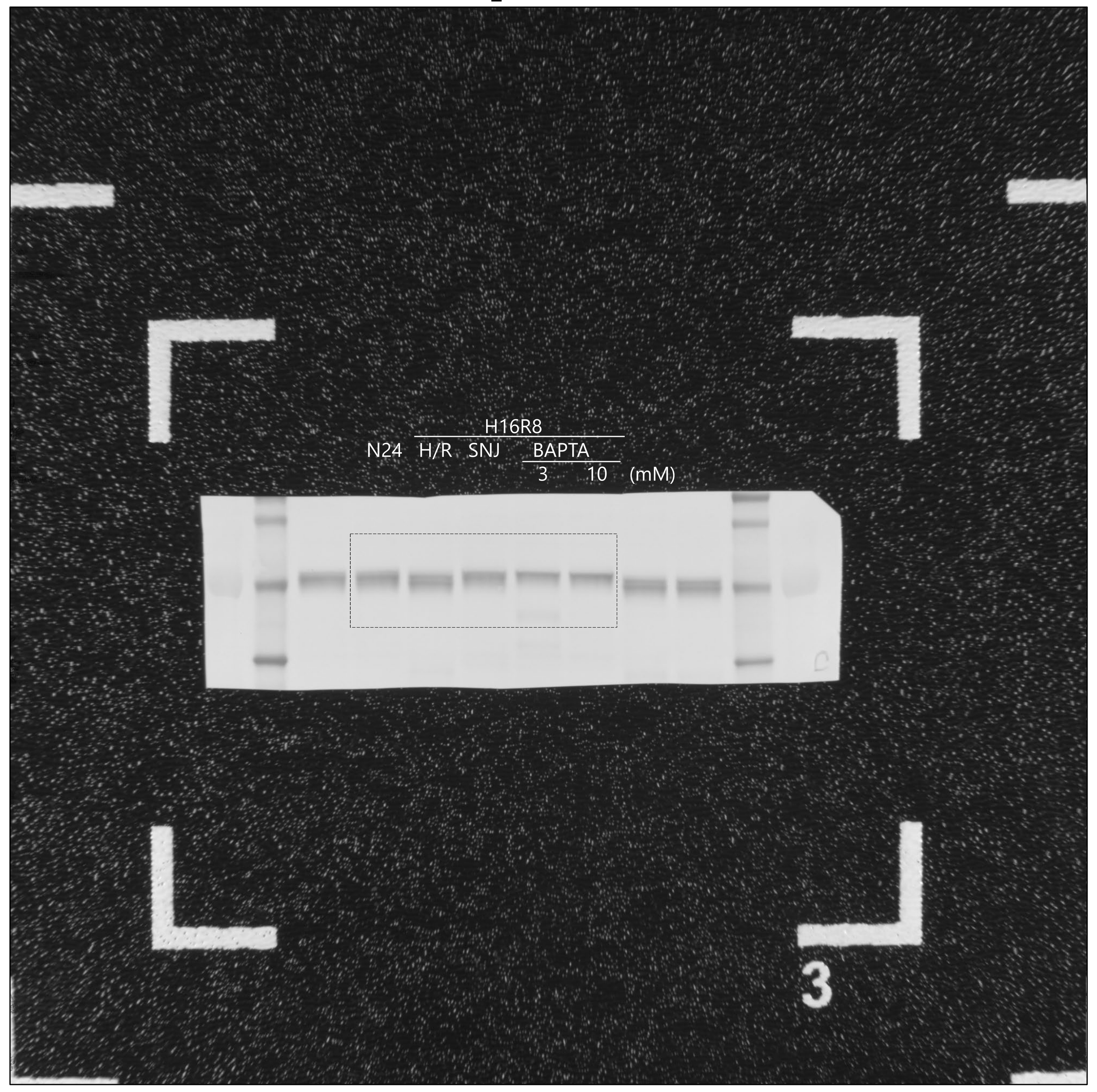

β-actin

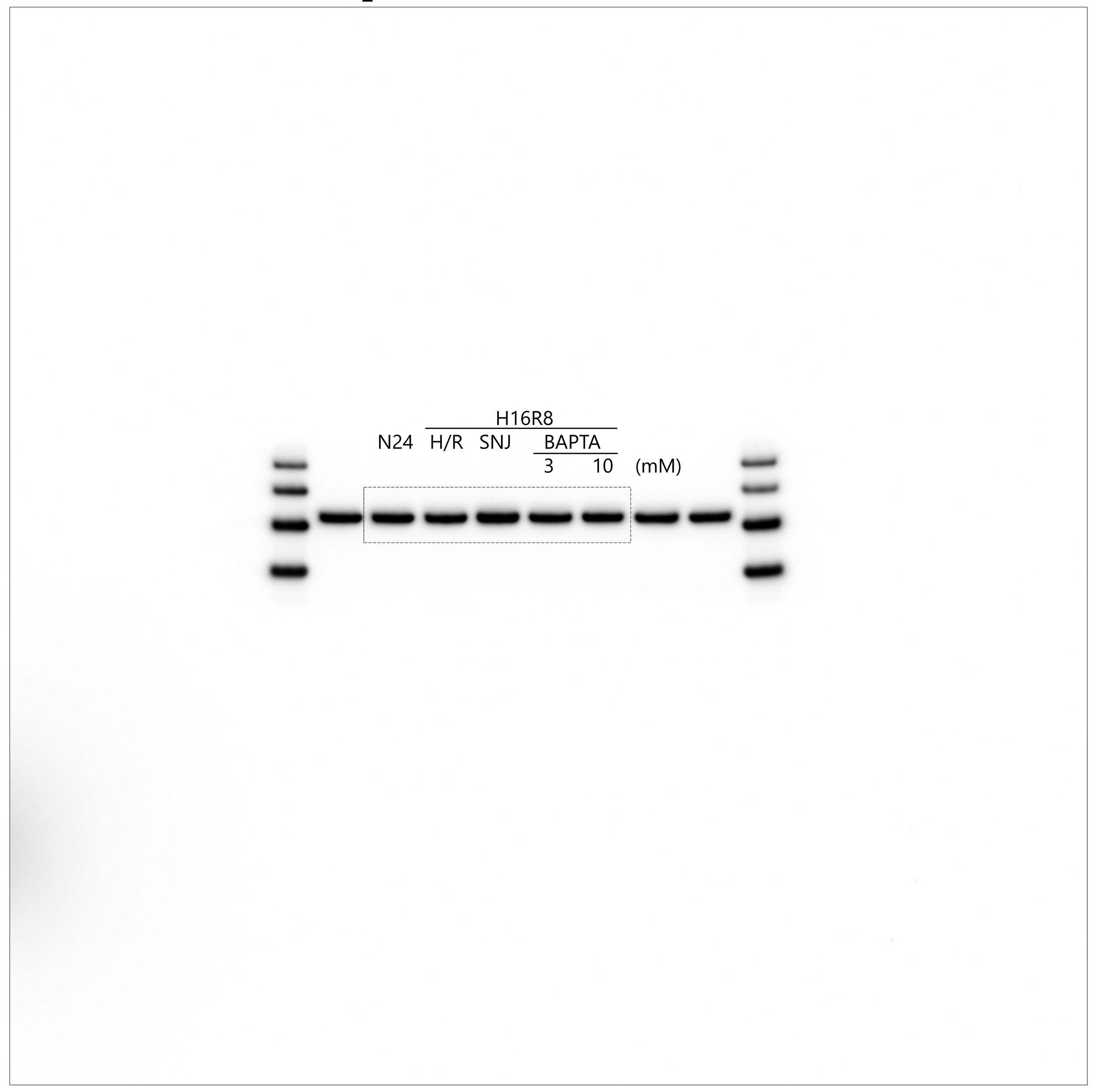

Original blot images of Fig3B

SBDP150

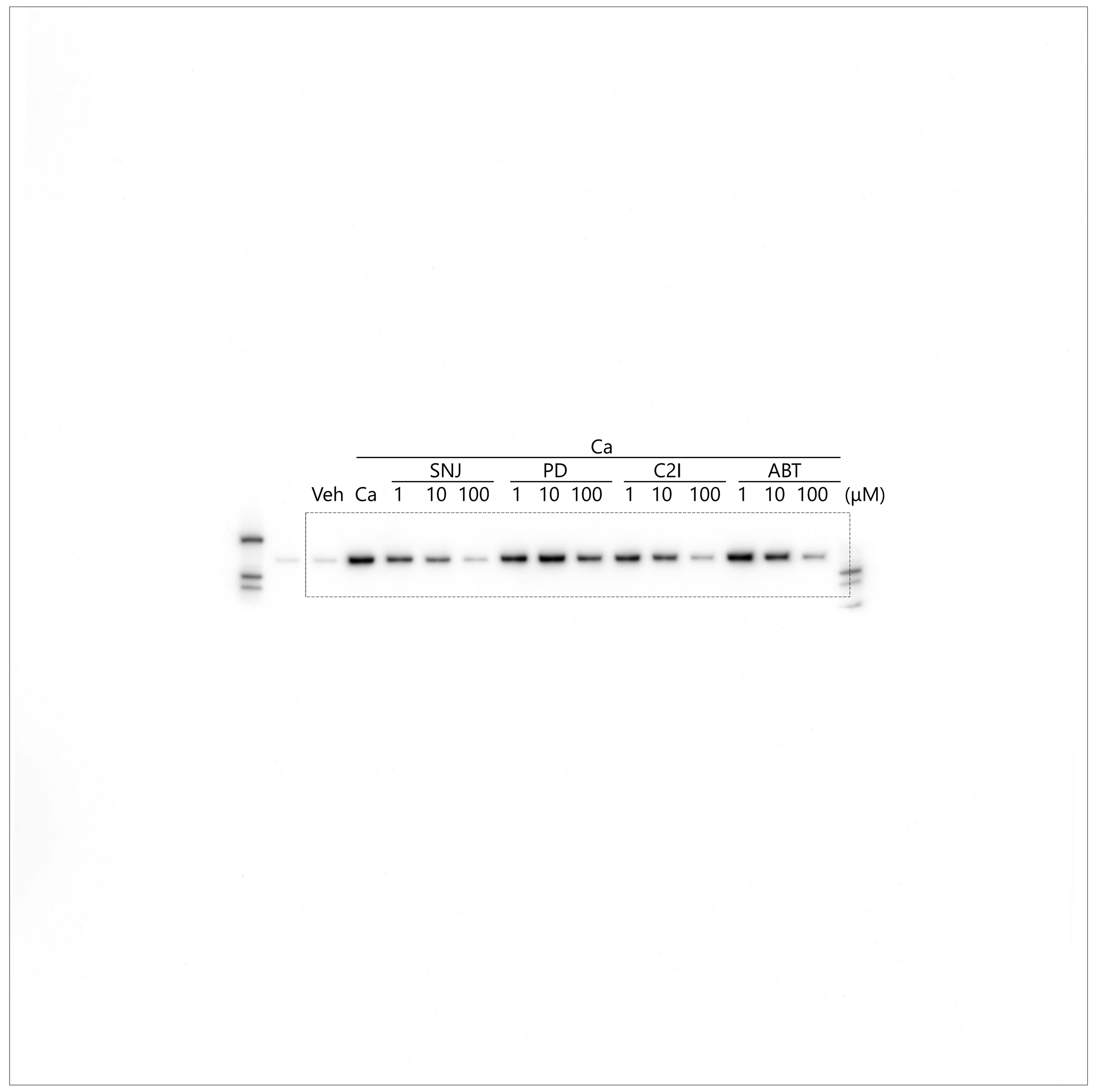

Calpain1

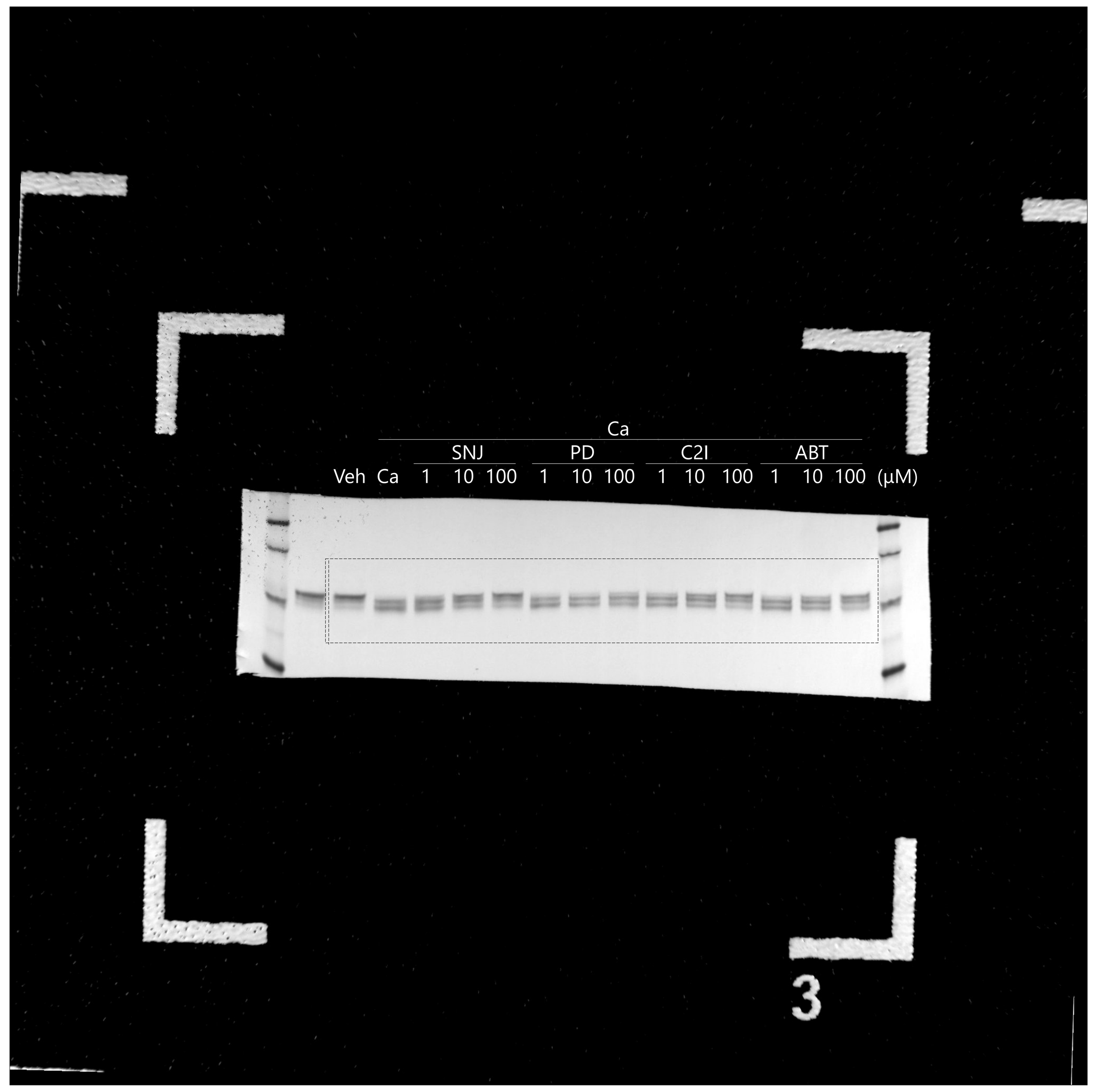

Calpain2

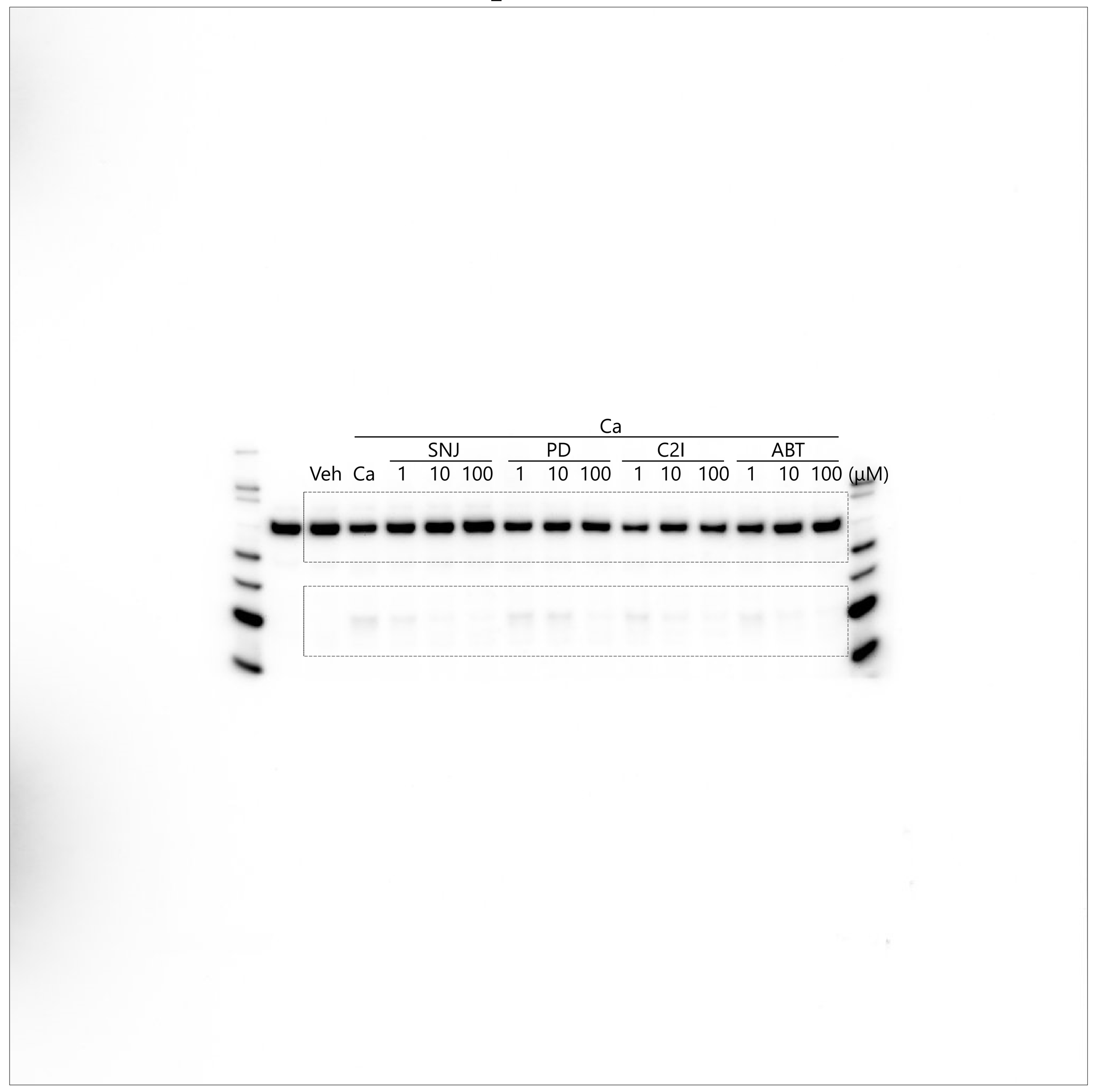

β-actin

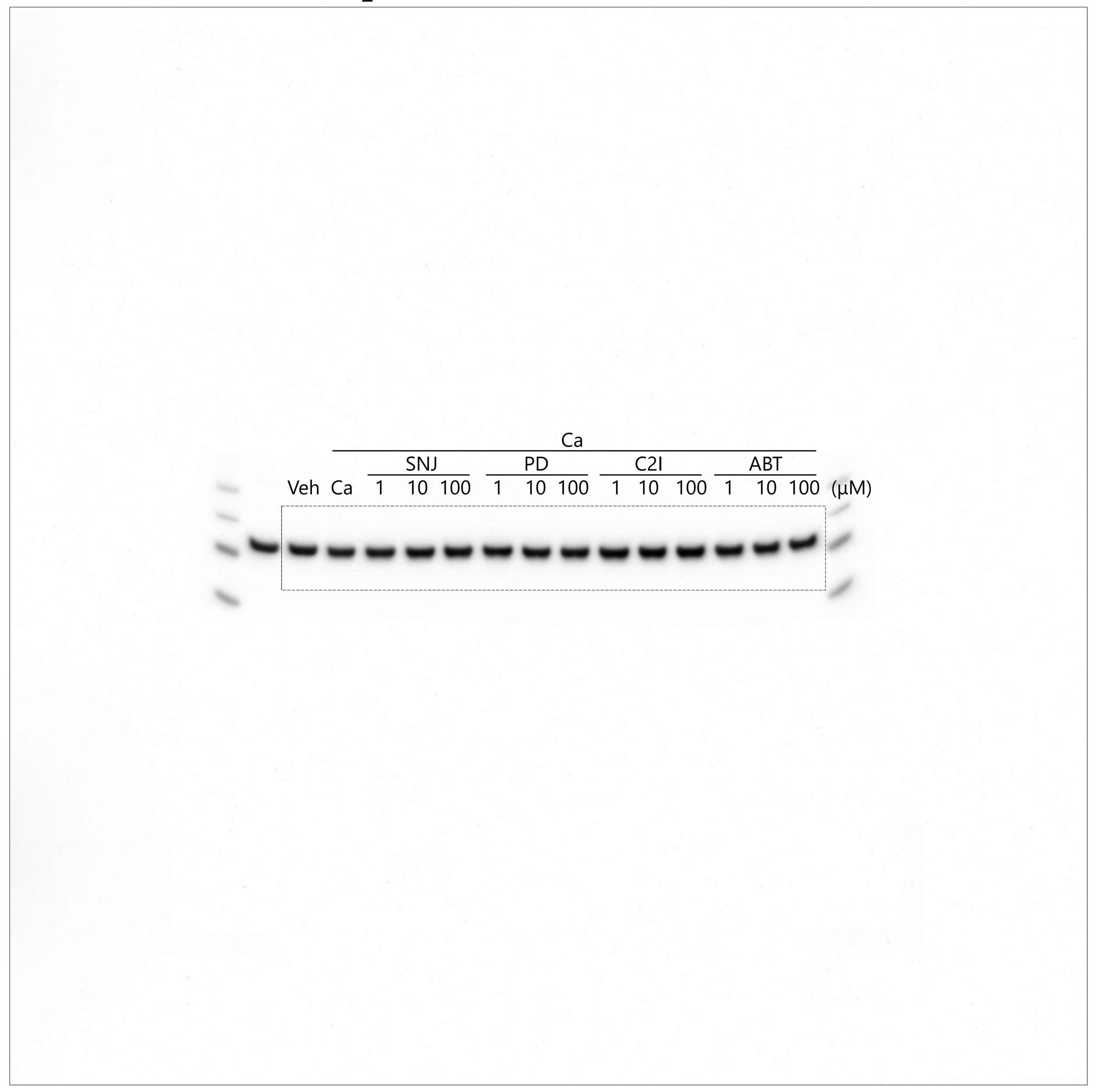

Original blot images of Fig3F

α-spectrin

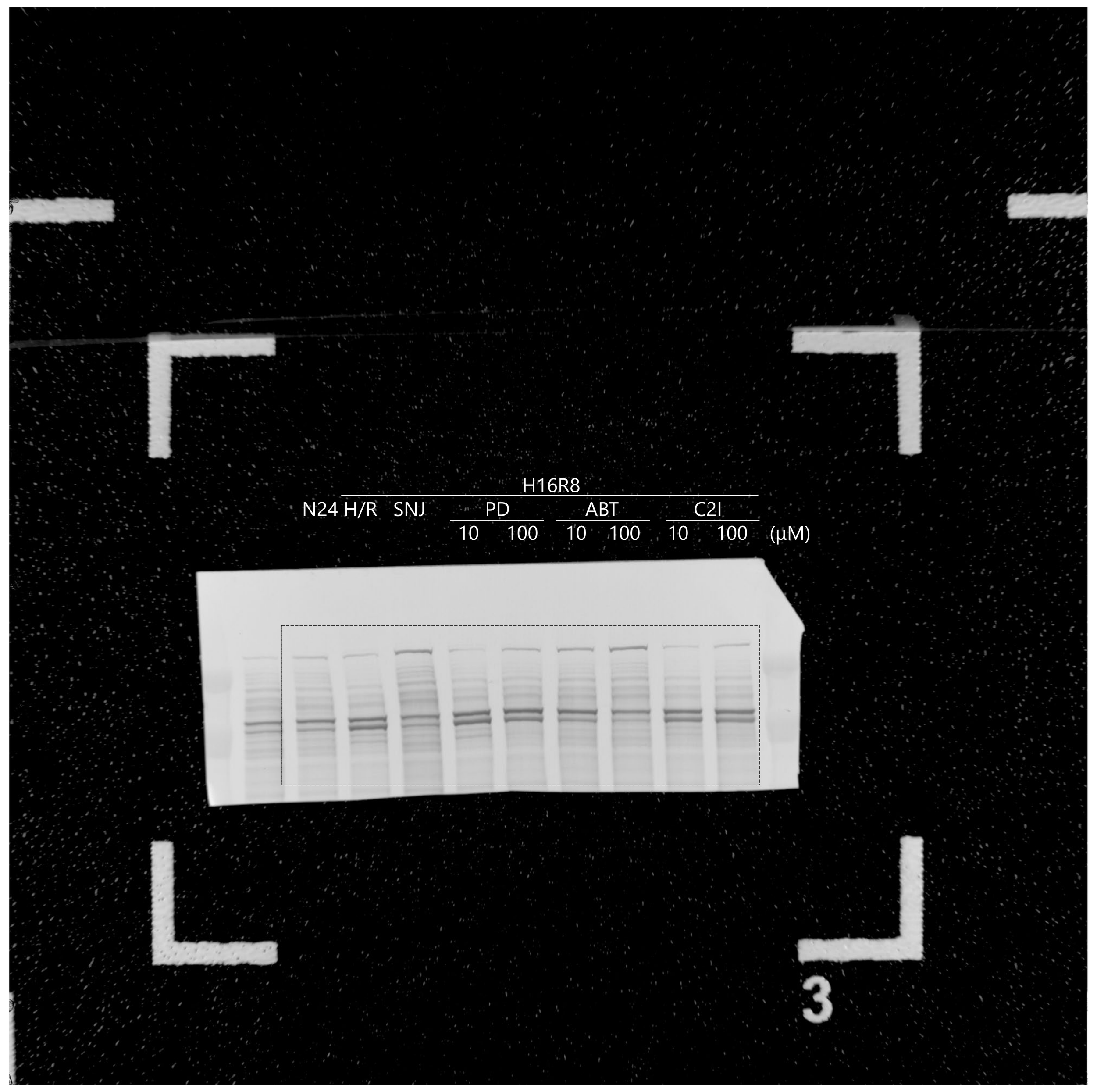

SBDP150

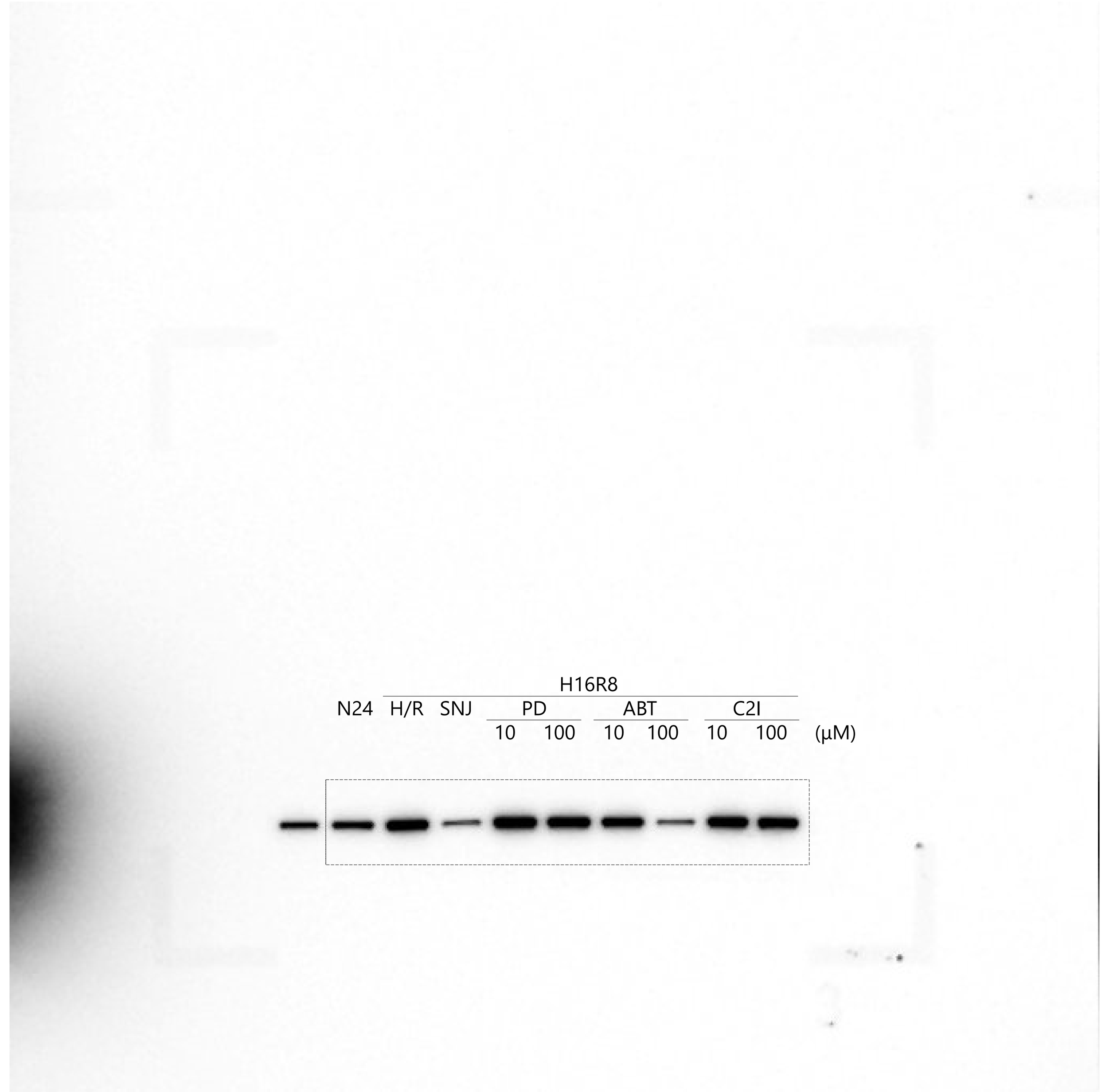

Calpain1

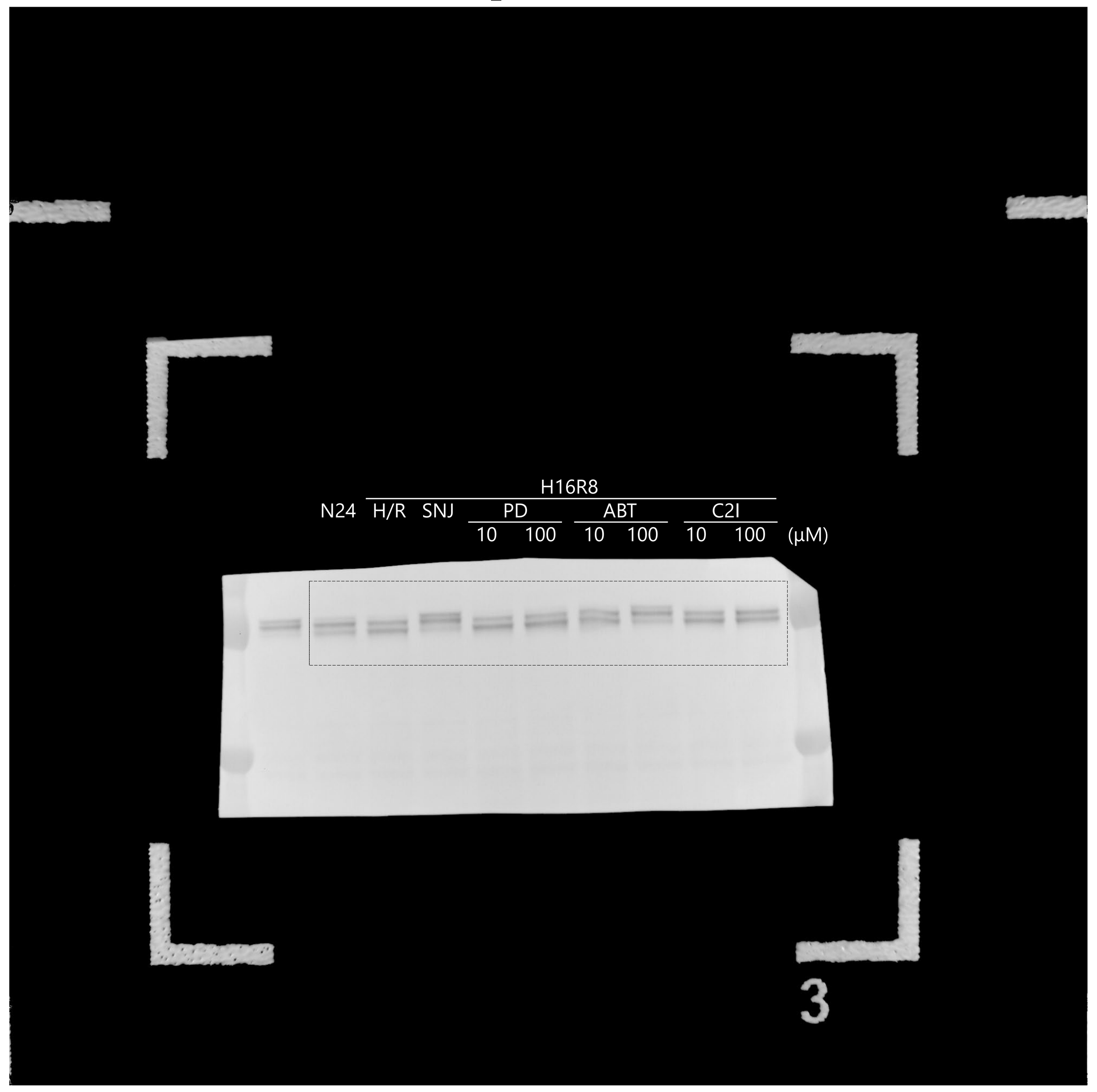

β-actin

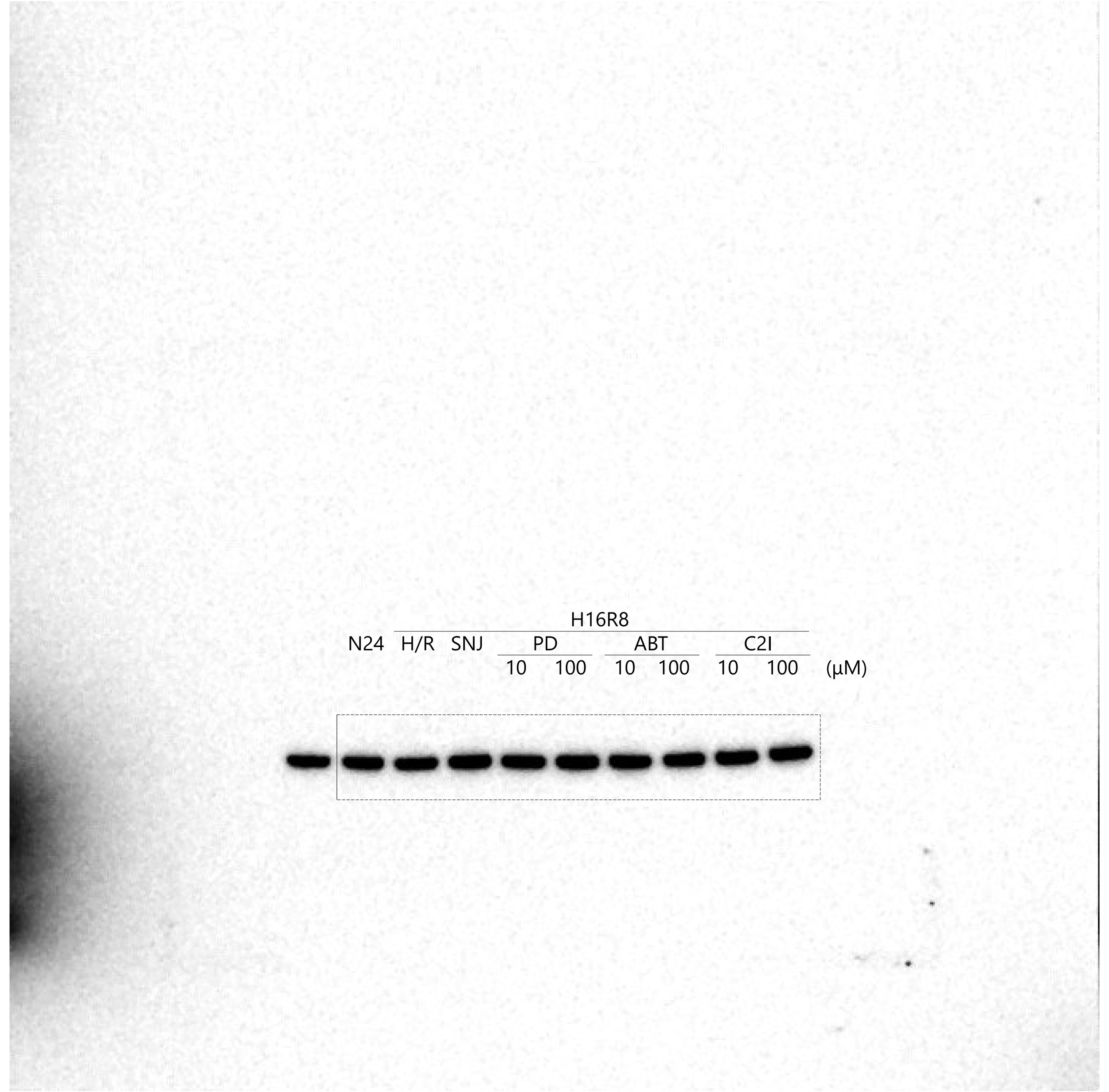

Supplement: S1 Fig — (PDF) [file pone.0327246.s001.pdf]
