# Supplementary material for: Calcium-dependent pathway as a primary cause of hypoxic RGC damage in monkey retinal explants
Source: PLoS One. 2025 Jul 11;20(7):e0327246. doi: 10.1371/journal.pone.0327246 (PMC12250347; doi:10.1371/journal.pone.0327246)
Supplement: Attachment — Submitted filename: Response to Reviewers.docx [file pone.0327246.s003.docx]

I have received your comments of our manuscript entitled “**Calcium-dependent pathway as a primary cause of hypoxic RGC damage in monkey retinal explants**.” We thank you to the reviewer for providing us with tremendous support to make our manuscript more accurate, clear and readable.

Reviewer's Comments to Author:

Reviewer #1: In the manuscript entitled ”Calcium-dependent pathway as a primary cause of hypoxic RGC damage in monkey retinal explants”, the authors primarily focused on retinal ganglion cell (RGC) damage, investigating both calcium-dependent and calcium-independent mechanisms involving calpain and cathepsins. This study builds upon the authors' previous work, where they observed the involvement of calpains. The current study focuses on whether RGC damage is mediated by calcium-dependent calpains, rather than by calcium-independent cathepsins.

Comments:

Given that the authors are investigating the involvement of calcium-independent cathepsins, could they clarify why experiments to measure the expression or presence of cathepsins in retinal lysates or flat mounts, as well as any associated quantitative analyses, were not performed in this study?

As noted in the Discussion, specific cleavage sites of α-spectrin by cathepsins have not been identified. Therefore, unlike calpain activation, α -spectrin cannot be used as a reliable marker for cathepsin activity. This limitation has now been stated in the revised Discussion.

Instead, we analyzed the conversion of pro-cathepsin K to its mature form as an indicator of cathepsin activation. Our results showed that hypoxia/reoxygenation did not induce the conversion of pro-cathepsin K to its mature form, in contrast to the activation observed for calpain 1. These findings have presented in Figure 1F.

However, it should be noted that only cathepsin K was examined in this study, although 11 cathepsins have been reported in the literature. This limitation has also been added to the revised Discussion.

Can the authors kindly provide the specific reference numbers for references 16-19 within the text, as they appear to be missing?

References 16 and 17 were used in the previous version, and we forgot to remove them when we revised the manuscript for submission. They are removed from the refence section, and the rest of the references were re-numbered. References 18 and 19 were re-numbered to references 14 and 15, respectively. We also found that references 23 and 24 were identical. Reference 24 was removed.

Considering that there are 11 types of cysteine cathepsins present, could the authors kindly elaborate on why they specifically chose to focus on cathepsins K, B, and L? Was there a particular rationale behind this selection?

The rationale for focusing on cathepsins K, B, and L lies in their well-documented involvement in cell death pathways and their early release following lysosomal membrane permeabilization. This explanation has now been included in the Materials and Methods section, along with appropriate citation (reference 13).

Line 255: Since the labeling in Figure 1 is not very clear, but assuming that SNJ treatment mitigated RGC death, could the authors please clarify why the reference is included there? What specific information or support do the authors intend to convey with this reference? It appears that references 21 and 22 are identical.

Figure 1B and 1C have been modified with clearer labeling. We hope this clarifies the mitigation of RGC death following SNJ-1945 treatment. Reference 21 was included because the effect of SNJ-1945 shown in Figure 1 was reproduced based on the findings reported in that reference. This has been clarified in line 258. As suggested by the reviewer, Reference 22 has been removed, as it was identical to Reference 21.

As mentioned in the Materials and Methods section, the authors did not provide any evidence or representative images demonstrating the activity of cathepsins and their effects on the substrate. Instead, the focus seems to be primarily on calpains. Could the authors clarify why the activity of cathepsins was not further investigated or visualized in this study?

As mentioned in the Materials and Methods section, the transformation of pro-cathepsin K to its mature form was used as a marker of cathepsin activation. This has now been clearly described in the revised manuscript. However, no such conversion was observed under our experimental conditions (Figure 1F), suggesting that cathepsin K was not activated.

Further investigation into cathepsin activity was not conducted in this study due to insufficient information, such as the lack of identified specific cleavage sites for cathepsins.

Instead, cathepsin inhibitors were used to gain partial insight into their potential role. However, these inhibitors did not suppress any of the changes induced by hypoxia/reoxygenation.

Taken together, these findings suggest that we were only able to partially evaluate cathepsin activity under the current conditions. We recognize the importance of further investigation, which will be considered in future studies. As explained in our responses to the previous comments, these points have now been incorporated into the revised manuscript.

Line 266: Although the labeling is not very clear, assuming that the third lane represents the treatment with SNJ, as indicated by the authors, there doesn't appear to be a decrease in SBDP150 levels. Could the authors kindly explain this discrepancy? Additionally, was any quantitative analysis performed to assess the changes in SBDP150 levels following treatments?

Labels for Fig. 1E and 1F have been modified with clearer labeling, and unnecessary lane 1 has been removed. We hope this clarifies the decrease in SBDP150 levels in the third lane of revised Figure 1E. As suggested by the reviewer, a graph showing the densitometric analysis has been added the blot image in Figure 1E to provide quantitative support for the observed changes.

In Figure 2a, the panel for H16R8 500 μM shows a decrease in RGC numbers. Although the pretreatment was administered 2 hours prior to hypoxic induction, could the authors discuss what might have caused the observed decrease in RGC numbers?

We would like to clarify that Figure 2a shows PI-positive RGCs, which represent damaged or dying cells. As the reviewer noted, treatment with 500 µM BAPTA-AM resulted in a decrease in the number of PI-positive RGCs following H16R8 exposure. This indicates that BAPTA-AM, when administered 2 hours prior to hypoxic induction, effectively mitigated RGC damage. Therefore, the observed decrease reflects a positive effect, rather than a loss of total RGCs.

Could the authors provide a label for calpain 2 in Figure 3?

Labels for Calpain 2 intact band and the cleaved band are now added in Figure 3B.
